# Estimating the Relative Contribution of Environmental and Genetic Risk Factors to Different Aging Traits by Combining Correlated Variables into Weighted Risk Scores

**DOI:** 10.3390/ijerph192416746

**Published:** 2022-12-13

**Authors:** Claudia Wigmann, Anke Hüls, Jean Krutmann, Tamara Schikowski

**Affiliations:** 1 IUF—Leibniz Research Institute for Environmental Medicine, 40225 Duesseldorf, Germany; 2 Department of Epidemiology, Rollins School of Public Health, Emory University, Atlanta, GA 30322, USA; 3 Gangarosa Department of Environmental Health, Rollins School of Public Health, Emory University, Atlanta, GA 30322, USA; 4 The Human Phenome Institute, Shanghai 200433, China

**Keywords:** aging, environmental exposure, exposome, relative contribution, relative importance, risk score

## Abstract

Genetic and exposomal factors contribute to the development of human aging. For example, genetic polymorphisms and exposure to environmental factors (air pollution, tobacco smoke, etc.) influence lung and skin aging traits. For prevention purposes it is highly desirable to know the extent to which each category of the exposome and genetic factors contribute to their development. Estimating such extents, however, is methodologically challenging, mainly because the predictors are often highly correlated. Tackling this challenge, this article proposes to use weighted risk scores to assess combined effects of categories of such predictors, and a measure of relative importance to quantify their relative contribution. The risk score weights are determined via regularized regression and the relative contributions are estimated by the proportion of explained variance in linear regression. This approach is applied to data from a cohort of elderly Caucasian women investigated in 2007–2010 by estimating the relative contribution of genetic and exposomal factors to skin and lung aging. Overall, the models explain 17% (95% CI: [9%, 28%]) of the outcome’s variance for skin aging and 23% ([11%, 34%]) for lung function parameters. For both aging traits, genetic factors make up the largest contribution. The proposed approach enables us to quantify and rank contributions of categories of exposomal and genetic factors to human aging traits and facilitates risk assessment related to common human diseases in general. Obtained rankings can aid political decision making, for example, by prioritizing protective measures such as limit values for certain exposures.

## 1. Introduction

Human phenotypes in general and health outcomes such as aging traits in particular result from genetic and non-genetic influences. For the latter the term exposome has been coined, which according to Christopher Wild is the totality of all non-genetic factors a human individual is exposed to from conception to death [1]. The exposome not only encompasses environmental factors but also lifestyle and behaviors, social environment and social status as well as the biological response [2]. 

The exposome concept is a step towards an all-embracing assessment of environment and health. It is potentially very interesting with regards to risk assessment, because knowledge about the relative contribution of distinct exposomal and genetic factors to a specific health outcome or aging trait would allow for more a precise and efficient prevention. 

However, to date it is still challenging to measure all exposures of the exposome continuously over the entire lifetime for each individual [3]. Apart from the difficulty in measuring the exposome, the statistical analysis is also not straightforward [3]. Even a cross-sectional analysis of the complex associations between different exposures, genetics and health outcomes is a statistical challenge, as many exposures are highly correlated. Epidemiological studies typically report exposure–health associations on an individual exposure-by-exposure basis while adjusting for confounding [3]. Correlated exposures are usually not included in traditional regression models due to concerns about multicollinearity. A recent review of statistical approaches used in the context of exposome studies is given in Guillien et al. (2021) [4]. Most of these approaches focus on the detection of causal exposures, i.e., variable selection, and on prediction of individual health status.

This article provides means for studying the contribution of a risk factor category such as air pollution as a whole, that is, the combined contribution of different exposures (e.g., nitrogen dioxide and particulate matter) belonging to the same category. The goal of the here-proposed methodology is to determine the extent to which each of several categories within the exposome as well as genetic factors contribute to a certain health outcome. Knowing these relative contributions would also enable comparisons between different populations and between different outcomes.

Since the concept of the exposome was introduced as a complement to the genome, methodology developed for genome-wide analyses might also be useful for analyses of the exposome. Consequently, exposome-wide association studies (in analogy to genome-wide association studies) have been proposed to assess the exposome [5]. These association studies use univariate single-exposure regression that does not take co-exposures into account and hence cannot provide a measure of relative contribution of a whole category of risk factors.

Approaches for multi-exposure regression like penalized regression models (LASSO [6] or elastic net [7]), the deletion/substitution/addition (DSA) algorithm [8], weighted quantile sum regression [9] (and its extension “quantile-based g-computation” [10]) or Bayesian kernel machine regression [11] take co-exposures into account, but are not useful for estimating the relative contributions of several risk factor categories either. Penalized regression and the DSA algorithm are variable selection methods and cannot directly infer the combined effects of categorized exposures. Weighted quantile sum regression and quantile-based g-computation combine several correlated exposures into one index and enable the estimation of the joint effect of an exposure mixture, but cannot handle categorical exposures. In addition, an extension for several risk factor categories of the exposome with more than one index remains unclear. Bayesian kernel machine regression can take hierarchical structures of the exposome into account by partitioning correlated exposures into several categories (“groups”) through a-priori information, but only a single exposure per category and not a mixture is allowed to contribute to the final estimates. In addition, this method is also not able to handle categorical exposures. 

A multivariate approach for genome-wide analyses is polygenic risk scores [12], which was developed for determining the genetic basis underlying a trait or disease, where the genetic predictors are in part highly correlated. Here, we will borrow the polygenic risk score methodology and adopt it to the exposome concept. Weighted risk scores, defined as weighted sums of all exposures belonging to the same risk factor category, will be constructed to analyze the combined impact of different (correlated) environmental exposures on health. More precisely, the proposed methodology consists of two steps: (1) combining correlated predictors into one weighted risk score (RS) per risk factor category and (2) estimating the relative contribution of each RS to a certain outcome using shares of explained variance in a linear regression model. The weights of the predictors will be determined internally in a training sample using regularized regression (explicitly, ridge regression) and a bootstrapping approach to account for the randomness in splitting the data set into training and test set. Estimating the weights by regularized regression in a training sample has been widely used for the construction of polygenic risk scores in general [13,14] and in the context of gene–environment–interaction studies [15,16,17]. In the second step, the Lindeman–Merenda–Gold measure of relative importance [18] is applied to assess the contribution of the composed risk scores to the health outcome in the test sample.

Here, data from the German SALIA cohort (Study on air pollution, lung function, inflammation and aging) is analyzed to prove the concept of applying risk scores to estimate relative contributions of environmental and genetic risk factors. The concept is applied to two aging traits, namely skin aging and aging-associated decreased lung function, to evaluate the relative contributions. The focus is on these two aging traits because for both it is well established that genetic and a number of specific exposomal factors contribute to their development [19,20]. Accordingly, important exposomal factors for lung aging are tobacco smoke and air pollution [20,21,22]; for skin aging these include exposure to ultraviolet (UV) radiation, air pollution and tobacco smoke, as well as nutritional factors [19].

## 2. Materials and Methods

**Figure 1:**
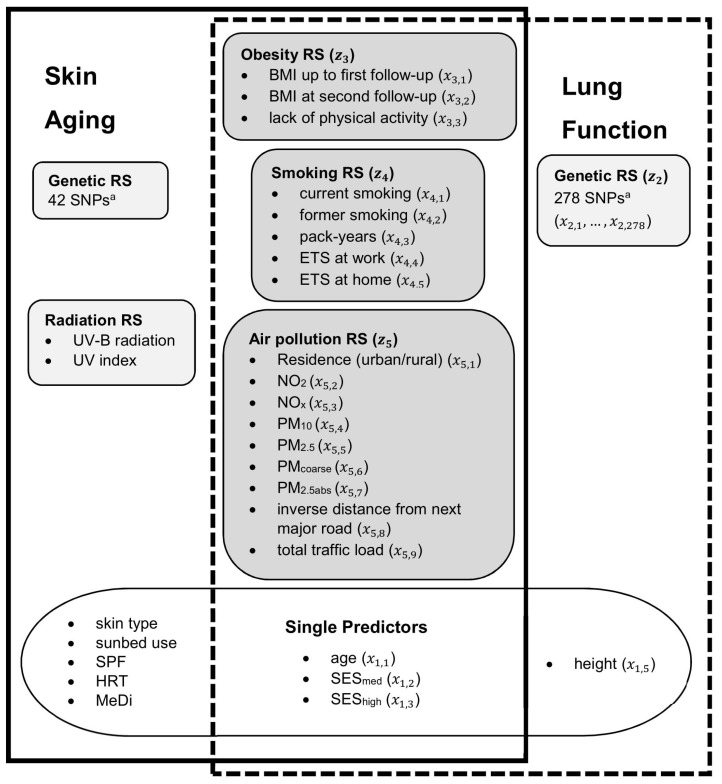
Overview of the risk scores and the combined predictors for both aging outcomes. The notation used in the description of the statistical approach is given in brackets, exemplary for lung function. BMI: body mass index; ETS: environmental tobacco smoke; HRT: hormone replacement therapy; MeDi: Mediterranean diet (definition: see Supplementary Materials); NO_2_: nitrogen dioxide; NO_x_: nitrogen oxides; PM_10_: particulate matter with aerodynamic diameter ≤ 10 µm; PM_2.5_: particulate matter with aerodynamic diameter ≤2.5 µm; PM_coarse_: coarse fraction of PM_10_ calculated as PM_10_ minus PM_2.5_; PM_2.5abs_: absorbance of particulate matter with aerodynamic diameter of ≤2.5 μm; RS: risk score; SES: socio-economic status; SNP: single nucleotide polymorphism; SPF: sun protection factor; UV: ultraviolet. ^a^ Details on the selected SNPs are given in Supplementary Tables S2 and S3 (Supplementary File S1).

The proposed methodology was applied to data from the SALIA cohort study of elderly German women by analyzing three aging traits: a z-score of facial pigment spots and z-scores of the lung function parameters Forced Expiratory Volume in 1 s (FEV_1_) and Forced Vital Capacity (FVC). Details on the study population [23,24,25] as well as outcome [26,27,28,29], genetic [30,31,32,33,34,35,36], exposure [37,38,39,40] and confounder variables can be found in the Supplementary Methods and Supplementary Tables S1–S3 (Supplementary File S1). An overview of the variables is given in Figure 1 and descriptive statistics can be found in Table 1. The de-correlating effect of building the risk scores is demonstrated with correlation plots in Supplementary Figures S1–S5 (Supplementary File S1).

### 2.1. Choice of Predictors

The risk scores combinepredefined predictors. In addition to the risk scores, further predictors are included as single predictors in the linear regression models. Figure 1 gives an overview for both the skin aging and the lung function analyses.

All predictors except the SNP variables have been standardized to mean zero and standard deviation one to achieve a fair penalization of all regressors in the following.

### 2.2. Bootstrap Data Sets and Division into Training and Test Sample

The following analysis steps are repeated *B* times using the bootstrap principle. That means that the analysis is not only conducted on the original data set, but also on *B-1* data sets created from the original data set by randomly sampling participants with replacement. Each bootstrap data set is then divided randomly into training and test samples in the relation 60% to 40%, as recommended in [12]. The number of bootstrap data sets is set to *B* = 500 for the skin aging outcome and *B* = 200 for the lung function outcomes, since in the latter case 278 SNP variables are included in the genetic RS resulting in considerably longer computation times.

### 2.3. Learning Risk Score Weights on Training Sample

The weights for the risk scores were learned on 60% of the participants in the training sample using ridge regression (implemented by elastic net regression with parameter α = 0), where the regularization parameter lambda was chosen via tenfold cross-validation with the R function cv.glmnet from R package glmnet [41]. Ridge regression was chosen, since variable selection was not the ultimate goal and shrinking all coefficients towards zero should retain the relations between the variables and lead to meaningful RS weights. The model formula, exemplary for the lung function analyses, is given by

$$y=\gamma_{0}+\underset{\mathrm{Single}\;\mathrm{predictors}}{\underbrace{\overset{4}{\underset{j=1}{\sum }}\gamma_{1,j\;}x_{1,j}}}+\underset{\mathrm{Genetic}\;\mathrm{predictors}}{\underbrace{\overset{278}{\underset{j=1}{\sum }}\gamma_{2,j\;}x_{2,j}}}+\underset{\mathrm{Obesity}\;\mathrm{predictors}}{\underbrace{\overset{3}{\underset{j=1}{\sum }}\gamma_{3,j}\;x_{3,j}}}+\underset{\mathrm{Smoking}\;\mathrm{predictors}}{\underbrace{\overset{5}{\underset{j=1}{\sum }}\gamma_{4,j}\;x_{4,j}}}+\underset{\mathrm{Air}\;\mathrm{pollution}\;\mathrm{predictors}}{\underbrace{\overset{9}{\underset{j=1}{\sum }}\gamma_{5,j}\;x_{5,j}}.}$$

The single predictors are included in this regression model to account for their effects on the outcome, but only the predictors belonging to the risk scores are regularized, since they are highly correlated and their coefficients will be used as weights in the risk scores. Since the folds for the cross-validation in cv.glmnet are selected at random, the results are random as well. To reduce this randomness, the estimation of the coefficients $\gamma_{k,j}$ is repeated twenty times and the resulting estimates are averaged. For each RS the respective subset of coefficients are normalized so that the resulting weights lie in $\left[-1,1\right]$ and sum to one for each RS. Explicitly, the weights of the risk scores are (exemplary for the lung function analyses) calculated as

$$w_{k,j}=\left\{\begin{array}{c} \frac{\hat{\gamma_{k,j}}}{\sum_{l=1}^{m_{k}}\left|\hat{\gamma_{l,j}}\right|},\;\;\exists \;j\;\hat{\gamma_{k,j}}\neq 0\\ \frac{1}{m_{k}},\;\;\hat{\gamma_{k,j}}=0\;\forall j=1,\cdots ,m_{k} \end{array}\right.,\;\;k=2,\cdots ,5$$

where $\hat{\gamma_{k,j}}$ are the estimates averaged over the twenty repetitions and $m_{k}$ is the number of predictors included in RS k (compare Figure 1).

### 2.4. Risk Scores, Linear Model and Relative Importance in the Test Sample

The RS weights derived from the training sample are used to calculate the values of each RS k in the test sample as the weighted average of the respective predictors:$\;z_{k}=\sum_{j=1}^{m_{k}}w_{k,j}\;x_{k,j},k=2,\cdots ,5$. The risk score values are then scaled by their interquartile ranges:$\;{\tilde{z}}_{k}=\frac{z_{k}}{IQR\left(z_{k}\right)},k=2,\cdots ,5$.

Afterwards, the risk scores and the fixed single predictors are used as independent variables in a multiple linear regression model for each outcome (here: a lung function index):

$$y=\beta_{0}+\underset{\mathrm{Single}\;\mathrm{predictors}}{\underbrace{\sum_{j=1}^{4}\beta_{1,j}\;z_{1,j}}}+\underset{\mathrm{Genetic}\;\mathrm{RS}}{\beta_{2}\underbrace{{\tilde{z}}_{2}}}+\underset{\mathrm{Obesity}\;\mathrm{RS}}{\beta_{3}\underbrace{{\tilde{z}}_{3}}}+\underset{\mathrm{Smoking}\;\mathrm{RS}}{\beta_{4}\underbrace{{\tilde{z}}_{4}}}+\underset{\mathrm{Air}\;\mathrm{pollution}\;\mathrm{RS}}{\beta_{5}\underbrace{{\tilde{z}}_{5}}}$$

Finally, the relative importance of all independent variables in this linear model is calculated with the R function calc.relimp [42], where the relative importance of socio-economic status (SES) is assessed using the two binary dummy variables as one group. The relative importance metric Lindeman–Merenda–Gold is used, which decomposes the coefficient of determination of the model, *R*^2^, by averaging sequential *R*^2^s over orderings of regressors [18, chapter 4.7]). The relative contributions given by this metric sum to the overall *R*^2^ [42]. 

All analysis steps are carried out for each bootstrap sample. The results presented in the following section are thus based on medians and percentiles across the bootstrap samples. The regression coefficients of the risk scores reflect the change in the outcome for an increase of one interquartile range of the respective RS and are used to determine significance of the association. Since the relative contributions of the risk scores are given as percentages, the corresponding bootstrap confidence intervals lie by definition above zero and cannot determine significance. All calculations and figures were done using R version 4.0.3 [43], except for Figure 1, which was produced using Microsoft Office Professional Plus 2019.

## 3. Results

### 3.1. Descriptive Analyses of the Outcomes and the Predictors

Descriptive statistics for all outcome and predictor variables are given in Table 1 and have been calculated for all 547 participants with available genetic and skin aging data and for 510 participants with available genetic and lung function data. 

The two analysis samples differ only slightly in their characteristics. The women were on average 73 years old at the time of the second follow-up, were on average mildly overweight (mean BMI: 27 kg/m^2^) and according to the mean MeDi score of 28.5 their nutritional habits moderately followed the Mediterranean diet. Only a few women were current or former smokers (2% and 16%, respectively), but about 33% were exposed to ETS at home and 42% at work.

### 3.2. Skin Aging Outcome

**Figure 2:**
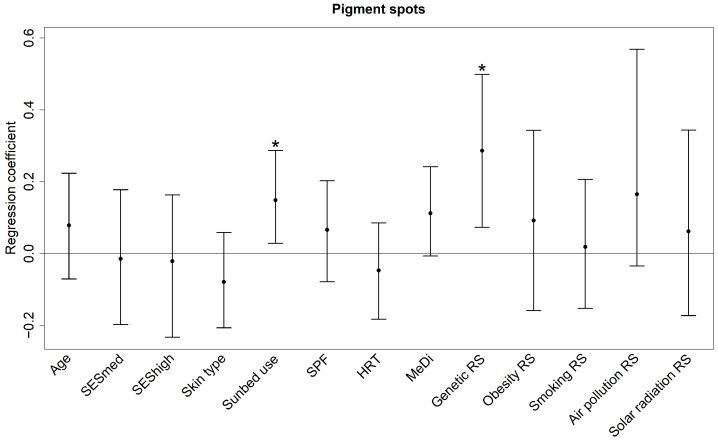
Regression coefficients (bootstrap medians and 95% percentile confidence intervals) of the predictors for pigment spots. The coefficients reflect the change in the z-score for one unit increase in the single predictors and one interquartile range increase in the risk scores. MeDi: Mediterranean diet; HRT: hormone replacement therapy; RS: risk score; SESmed: medium socio-economic status (reference: low socio-economic status); SEShigh: high socio-economic status (reference: low socio-economic status); SPF: sun protection factor; * *p* < 0.05.

For the outcome of facial pigment spots all predictors combined explain 16.90% of the variance (median total *R*^2^, Table 2). As can be seen from the regression coefficients in Figure 2, the genetic RS and sunbed use are associated with the formation of facial pigment spots (bootstrap medians, 95% percentile CIs and *p*-values: 0.29 [0.07, 0.50], *p* = 0.016; 0.15 [0.03, 0.29], *p* = 0.016) with the two highest median relative contributions of 4.15% and 2.11%. 

The contributions of MeDi (1.20%) and air pollution RS (1.14%) seem interesting for future research according to their regression coefficients (0.11 [−0.01, 0.24], *p* = 0.064 and 0.16 [−0.04, 0.57], *p* = 0.132) whereby it is noticeable that stronger adherence to the Mediterranean diet seems to increase the formation of pigment spots.

### 3.3. Lung Function Outcomes

**Figure 3:**
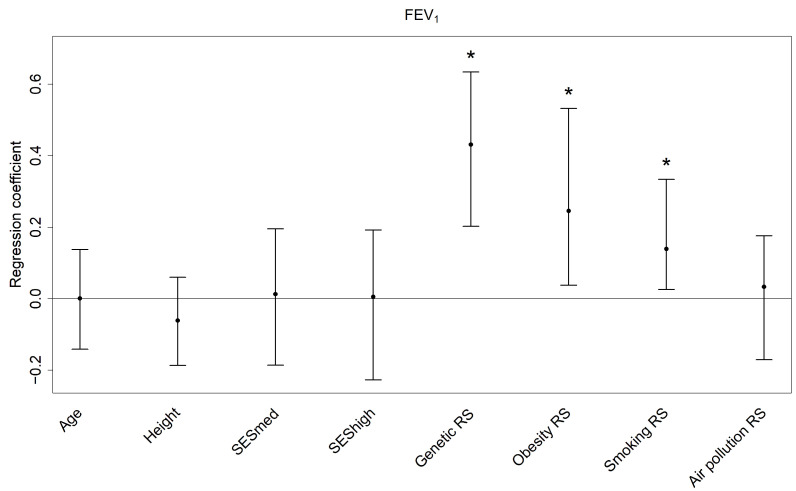
Regression coefficients (bootstrap median and 95% confidence interval) of the predictors for FEV_1_. The coefficients reflect the change in the z-score for one unit increase in the single predictors and one interquartile range increase in the risk scores. FEV_1_: forced expiratory volume in 1 s; RS: risk score; SESmed: medium socio-economic status (reference: low socio-economic status); SEShigh: high socio-economic status (reference: low socio-economic status); * *p* < 0.05.

**Figure 4:**
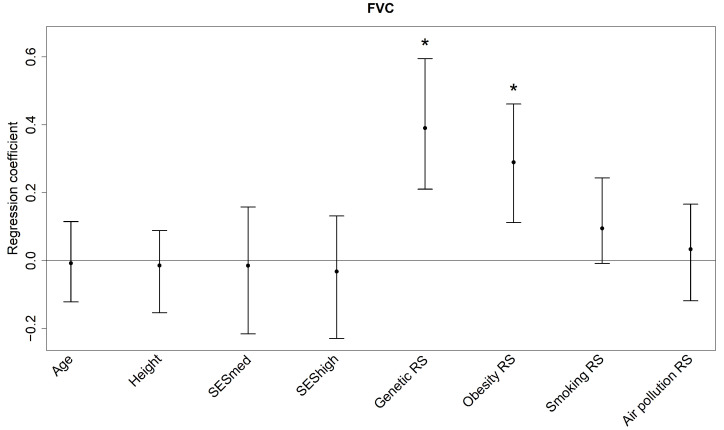
Regression coefficients (bootstrap median and 95% confidence interval) of the predictors for FVC. The coefficients reflect the change in the z-score for one unit increase in the single Predictors and one Interquartile Range Increase in the Risk Scores. FVC: forced vital capacity; RS: risk score; SESmed: medium socio-economic status (reference: low socio-economic status); SEShigh: high socio-economic status (reference: low socio-economic status); * *p* < 0.05.

All single predictors and risk scores combined explain in median 22.32% of the variance in FEV_1_ and 23.36% of the variance in FVC (see Table 3 and Table 4). The genetic RS is associated with both lung function parameters according to its regression coefficients (see Figure 3 and Figure 4; 0.43 [0.20, 0.63], *p* = 0.01 for FEV_1_ and 0.39 [0.21, 0.59], *p* = 0.01 for FVC) with relative contributions of about 11%. The risk scores for genetics, smoking and obesity are among the top three relative contributors to both outcomes. For FEV_1_ the obesity (median relative contribution 2.55%) and smoking risk scores (median relative contribution 4.98%) are additionally associated (0.25 [0.04, 0.53], *p* = 0.01 and 0.14 [0.03, 0.33], *p* = 0.00), while for FVC it is only the obesity RS (median relative contribution 5.56%; coefficient 0.29 [0.11, 0.46], *p* = 0.00). The smoking risk score’s bootstrap median coefficient for FVC is 0.09 with 95% percentile confidence interval [−0.01, 0.24], *p* = 0.06. It might seem as if the effects of obesity and smoking are beneficial due to the positive regression coefficients, but the risk scores’ weights are mostly negative so that a larger RS refers to less obesity and less tobacco smoke exposure.

## 4. Discussion

The proposed approach enables us to quantify the contributions of genetic factors and various categories of the exposome to a certain outcome in terms of percentages of explained variance, where each category has been assessed by several (correlated) variables and combined into one weighted RS. 

In these examples, the highest contribution to all aging traits was achieved by the genetic risk scores comprising the considered SNPs. Apart from the genetic and the obesity RS and in parts the smoking RS, the environmental risk scores were not associated with the outcomes. That the lower limit of the confidence interval of the air pollution risk score’s coefficient is very close to zero in the skin aging example is in line with previous analyses in the SALIA study, which found associations of air pollution with skin aging [44] when using single-pollutant models with no need to split off a training sample. One might have expected to see associations of the aging traits with age (at least for the skin aging outcome, where the z-score does not account for age). This is probably not only due to the small sample size, but also due to a small age range of the study participants.

The magnitudes and rankings of the estimated relative importance are quite similar compared between the two lung function parameters, while there are distinct differences when comparing the same predictors and risk scores between skin aging and lung function. The percentage of variance explained by the genetic RS in the skin aging outcome is only less than half of that in the lung function outcomes. In addition, the smoking risk score’s relative contribution, for example, ranks very low for skin aging, while it has a top three ranking for lung function. Thus, awareness campaigns and other measures to reduce the number of smokers seem more important for reducing lung aging than for delaying skin aging in the population. Against skin aging it would be more effective to inform about the negative effects of sunbed use.

Overall, the models were able to explain between 17% and 23% of the different outcomes’ variances, which is noticeable considering the complexity of the aging processes, the limited sample size and the fact that further components of the exposome such as stress or lack of sleep which were not collected in the SALIA study could not be incorporated. Though the presented example is far from a complete exposome analysis, this investigation shows that (i) in principle the proposed approach can quantify the extent to which each of the various categories of the exposome contribute, and that (ii) these relative contributions vary for different health traits and thus can be ranked.

The approach has some limitations. First, it relies on the calculation of relative contributions via shares of explained variance in a linear regression model and is as a consequence limited to linear regression. Generalized linear models such as logistic regression for binary traits are not applicable, since the concept of relative contribution is not (easily) generalizable. This direction is interesting for future research since binary health outcomes are very common. Second, splitting the available data set into training and test samples does not only reduce statistical power in the linear regression analysis, but also requires repeated execution of the fitting process (here: several bootstrap samples) to reduce the randomness of the partition and the results. This, however, complicates reporting of the results which have to be averaged. In particular, residual diagnostics are not easily applicable since they would have to be examined for each bootstrap sample. Yet, there is usually no alternative to internal weights for the risk scores, since external weights from published studies with several covariates belonging to the same risk factor category are typically not available precisely because they are often highly correlated. Third, combining several covariates in one (weighted) RS certainly leads to loss of information. An alternative would be to directly report the results of a regularized regression without the formation of risk scores. However, the aim of this study was to show to which extent each risk factor category contributes to the outcome. To the best of our knowledge, the concept of relative contribution is not (yet) applicable to regularized regression models. Such an extension is interesting for future research. In addition, this study is limited to a certain configuration of model parameters. For example, there might be choices other than α = 0 (ridge regression) in the elastic net or other regularized regression or machine learning methods, which yield more appropriate RS weights for our purposes, but a comparison is beyond the scope of this work. Nevertheless, the presented application and results provide a proof of concept for the proposed methodology.

## 5. Conclusions

The combination of risk scores with a measure of relative contribution is suitable to assess the extent to which various categories of the exposome and genetic factors contribute to a certain health outcome, and the contributions can not only be compared between different outcomes, but also between, for example, different ethnic or age groups. In addition, the exposome’s categories can be ranked according to their relative contribution. The proposed approach might thus have the potential to improve risk assessment relevant for human aging traits and beyond, i.e., common human diseases. In this regard it could be of interest not only to health scientists, but also to governmental institutions, because it might help to prioritize regulatory decisions limiting exposure to selected environmental factors and put them on a more solid scientific basis.

## Figures and Tables

**Table 1:** Descriptive statistics of all outcome and predictor variables in both analysis samples.

Variable	Categories	Mean (SD) or *n* (%)	
		Skin aging analysis (*n* = 547)	Lung function analysis (*n* = 510)
Outcomes			
Pigment spot z-score		0.00 (1.00)	
FEV_1_ z-score			0.17 (1.02)
FVC z-score			0.24 (0.90)
FEV_1_/FVC z-score			−0.22 (0.85)
Single Predictors			
Age [years]		73.58 (2.97)	73.52 (2.99)
Height [cm]			162.83 (5.76)
SES:	1 = “<10 years”	94 (17.2%)	89 (17.5%)
	2 = “10 years”	275 (50.3%)	249 (48.8%)
	3 = “>10 years”	178 (32.5%)	172 (33.7%)
Skin type:	dark (0; Fitzpatrick type 3 and 4)	243 (44.4%)	
	light (1; Fitzpatrick type 1 and 2)	304 (55.6%)	
Sunbed use:	never (0)	459 (83.9%)	
	ever (1)	88 (16.1%)	
SPF:	no (0)	214 (39.1%)	
	yes (1)	333 (60.9%)	
HRT:	no (0)	319 (58.3%)	
	yes (1)	228 (41.7%)	
MeDi score		28.50 (2.79)	
Obesity Risk Score			
mean BMI up to FU 1 [kg/m^2^]		26.70 (3.67)	26.66 (3.66)
BMI FU 2 [kg/m^2^]		27.32 (4.34)	27.29 (4.34)
Lack of physical activity:	no (0)	216 (39.5%)	203 (39.8%)
	yes (1)	331 (60.5%)	307 (60.2%)
Smoking Risk Score			
Current smoking:	no (0)	535 (97.8%)	498 (97.6%)
	yes (1)	12 (2.2%)	12 (2.4%)
Former smoking:	no (0)	458 (83.7%)	425 (83.3%)
	yes (1)	89 (16.3%)	85 (16.7%)
ETS at work:	never (0)	314 (57.4%)	294 (57.6%)
	ever (1)	233 (42.6%)	216 (42.4%)
ETS at home:	never (0)	365 (66.7%)	337 (66.1%)
	ever (1)	182 (33.3%)	173 (33.9%)
Packyears [packs/day × years]		3.68 (12.46)	3.83 (12.74)
Air pollution Risk Score			
Residence:	rural (0)	260 (47.5%)	249 (48.8%)
	urban (1)	287 (52.5%)	261 (51.2%)
NO_2_ [µg/m^3^]		37.73 (11.46)	37.21 (11.16)
NO_x_ [µg/m^3^]		70.52 (32.66)	69.17 (31.93)
PM_10_ [µg/m^3^]		49.27 (7.17)	48.98 (7.38)
PM_2.5_ [µg/m^3^]		32.75 (4.65)	32.56 (4.78)
PM_coarse_ [µg/m^3^]		17.57 (3.85)	17.42 (3.91)
PM_2.5abs_ [10^−5^/m]		2.74 (0.92)	2.71 (0.92)
Trafloadmajor [1000 vehicles × m/day]		900.63 (2313.15)	839.51 (2200.63)
Invdistmajor [1/m]		0.01 (0.02)	0.01 (0.02)
Radiation Risk Score			
UV-B [J/m^2^]		3140.38 (33.03)	
UV index [40 W/m^2^]		7.18 (0.09)	

BMI: body mass index; ETS: environmental tobacco smoke; FEV_1_: forced expiratory volume in 1 s; FVC: forced vital capacity; FU: follow-up; HRT: hormone replacement therapy; Invdistmajor: inverse distance to next major road (>5000 vehicles/day); MeDi: Mediterranean diet (definition: see Supplementary Materials); n: number of samples; NO_2_: nitrogen dioxide; NO_x_: nitrogen oxides; PM_10_: particulate matter with aerodynamic diameter ≤10 µm, PM_2.5_: particulate matter with aerodynamic diameter ≤2.5 µm; PM_coarse_: coarse fraction of PM_10_ calculated as PM_10_ minus PM_2.5_; PM_2.5abs_: absorbance of particulate matter with aerodynamic diameter of ≤2.5 μm; SD: standard deviation; SES: socio-economic status; SPF: sun protection factor; Trafloadmajor: total traffic load (number of vehicles/day × length of road segments) from major roads (>5000 vehicles/day) within 100 m buffer; UV: ultraviolet.

**Table 2:** Relative Contributions (bootstrap median and 95% percentile confidence interval) of the predictors for pigment spots, as percentages, sorted according to the median.

Predictor	Median	95% Confidence Interval
*Overall*	*16.90%*	*8.75%*, *28.04%*
Genetic RS	4.15%	0.25%, 11.88%
Sunbed use	2.11%	0.09%, 6.97%
Air pollution RS	1.20%	0.04%, 5.76%
MeDi	1.14%	0.04%, 5.11%
SES	0.70%	0.09%, 3.44%
Obesity RS	0.66%	0.04%, 4.59%
SPF	0.59%	0.04%, 3.33%
Skin type	0.58%	0.03%, 3.51%
Age	0.52%	0.04%, 3.37%
Solar radiation RS	0.45%	0.04%, 3.35%
HRT	0.29%	0.03%, 2.41%
Smoking RS	0.29%	0.02%, 2.74%

MeDi: Mediterranean diet; HRT: hormone replacement therapy; RS: risk score; SES: socio-economic status; SPF: sun protection factor.

**Table 3:** Relative Contributions (bootstrap median and 95% percentile confidence interval) of the predictors for FEV_1_, as percentages, sorted according to the median.

Predictor	Median	95% Confidence Interval
*Overall*	*22.32%*	*10.87%*, *33.57%*
Genetic RS	10.99%	2.92%, 23.29%
Smoking RS	5.10%	1.15%, 11.87%
Obesity RS	2.41%	0.17%, 7.62%
SES	0.80%	0.13%, 3.48%
Height	0.45%	0.02%, 2.92%
Air pollution RS	0.36%	0.02%, 2.69%
Age	0.26%	0.04%, 1.85%

FEV_1_: forced expiratory volume in 1 s; RS: risk score; SES: socio-economic status.

**Table 4:** Relative Contributions (bootstrap median and 95% percentile confidence interval) of the predictors for FVC, as percentages, sorted according to the median.

Predictor	Median	95% Confidence Interval
*Overall*	*23.36%*	*10.86%*, *33.67%*
Genetic RS	11.69%	2.80%, 25.11%
Obesity RS	5.62%	1.21%, 12.50%
Smoking RS	1.83%	0.06%, 6.42%
SES	0.90%	0.13%, 3.71%
Air pollution RS	0.42%	0.03%, 3.40%
Age	0.26%	0.03%, 2.58%
Height	0.24%	0.02%, 1.96%

FVC: forced vital capacity; RS: risk score; SES: socio-economic status.

## Data Availability

Due to privacy laws in Germany the data of the cohort study are not available. The R code can be obtained by contacting the authors of the paper.

## Supplementary Materials

The following supporting information can be downloaded at: https://www.mdpi.com/article/10.3390/ijerph192416746/s1, Supplementary File S1: Table S1: Scoring of the food frequencies for the Mediterranean diet score; Table S2: Single nucleotide polymorphisms used in the genetic risk score for the skin aging trait; Table S3: Single nucleotide polymorphisms used in the genetic risk score for the lung function traits; Figure S1: Correlation plot of the original variables used in the skin aging analysis (excluding the SNP variables); Figure S2: Correlation plot of the original variables used in the lung function analysis (excluding the SNP variables); Figure S3: Correlation plot of the risk scores and single predictors used in the skin aging analysis; Figure S4: Correlation plot of the risk scores and single predictors used in the analysis of FEV1; Figure S5: Correlation plot of the risk scores and single predictors used in the analysis of FVC. Refs. [23,24,25,26,27,28,29,30,31,32,33,34,35,36,37,38,39,40,45,46,47] are cited in the supplementary files.
