# Supplementary material for: Estimating the Relative Contribution of Environmental and Genetic Risk Factors to Different Aging Traits by Combining Correlated Variables into Weighted Risk Scores
Source: Int J Environ Res Public Health. 2022 Dec 13;19(24):16746. doi: 10.3390/ijerph192416746 (PMC9779342; doi:10.3390/ijerph192416746)
Supplement: Supplementary file 1 [file ijerph-19-16746-s001.zip › ijerph-2054533-supplementary.pdf]

Supplementary Material

for

“Estimating the relative contribution of environmental and genetic risk factors to different aging traits by combining correlated variables into weighted risk scores”

Authors:
Claudia Wigmann
Anke Hüls
Jean Krutmann
Tamara Schikowski*, IUF – Leibniz Research Institute for Environmental Medicine, Auf'm Hennekamp 50, 40225 Duesseldorf, Germany. E-mail: tamara.schikowski@iuf-duesseldorf.de

*Corresponding Author

Supplementary Methods

Study population

We demonstrate our methodology by applying the relative importance calculation on a subset of a cohort study in North Rhine-Westphalia, Germany. The Study on air pollution, lung function, inflammation and aging (SALIA) has been described in detail elsewhere [1,2]. Briefly, the baseline investigation (1985-1994) recruited *n* = 4874 55-year-old women from urban as well as rural areas (Ruhr area and Münsterland). The first questionnaire follow-up took place in 2006-2007. Here, we use mainly data from the second follow-up investigation (2007-2010), which examined *n* = 834 women including skin aging assessment, lung function testing, and extensive interview [3]. In addition, air pollution exposure, traffic indicators and ultraviolet (UV) radiation exposure were assigned to each participant's home address. Genome-wide single nucleotide polymorphism (SNP) data was collected for 586 women.

Outcome variables

Skin aging was assessed using the SCINEXA™ SCore for INtrinsic and EXtrinsic skin Aging [4]. For our analyses, we use a composite score (z-score) for facial pigment spots as the outcome.

Facial pigment spots were evaluated by number (categories 0: 0 spots, 1: 1-10 spots, 2: 11-50 spots, 3: >50 spots) and size (photoreference scales from "0: not present" to "5: very severely present" according to Tschachler [5]). The z-score for facial pigment spots is the unit-weighted mean of the z-transformed (minus mean and divided by standard deviation) skin aging traits "number of pigment spots on forehead", "size of pigment spots on the forehead", "number of pigment spots on cheeks" and "size of pigment spots on cheeks".

Lung function testing included spirometry measurements of Forced Expiratory Volume in 1 second (FEV_1) and Forced Vital Capacity (FVC) according to the guidelines of the American Thoracic Society and European Respiratory Society [6]. The lung function parameters were transformed to z-scores to account for effects of age, sex, and height using the Global Lung Initiative reference set [7]. Participants were instructed not to be smoking before the testing and were not tested when having an upper respiratory tract infection.

Exposure assessment

Air pollution exposures and traffic indicators were assessed according to the European Study of Cohorts for Air Pollution Effects (ESCAPE) protocol [8,9]. Exposure concentrations of nitrogen dioxide (NO_2), nitrogen oxides (NO_x), particulate matter with aerodynamic diameter less or equal 10 µm (PM_{10}) and 2.5 µm ($PM_{2.5}$), coarse fraction of PM_{10} (PM_{coarse}) calculated as PM_{10} minus $PM_{2.5}$ as well as absorbance of particulate matter with aerodynamic diameter less or equal 2.5 µm ($PM_{2.5abs}$) were assigned to the participants' home addresses at baseline using the ESCAPE land use regression model (which was for 2008-2009). These were backextrapolated to the baseline by the ratio method. A detailed description of the backextrapolation method can be found in [10]. The inverse distance from the home address to the next major road (>5000 vehicles / day) as well as the total traffic load (number of vehicles / day) from major roads within a 100m buffer around the residence were estimated as part of the ESCAPE project by using digital road network linked with traffic intensity data in a Geographic Information System.

UV radiation was assessed at the follow-up residential addresses as the 3-year average of UV-B radiation and UV index during summer months of 2004 to 2006 preceding the follow-up investigations. The UV-B dose (J/m^2) is based on the whole

daylight period. The UV index (1 unit corresponds to 40 W/m^2), which includes UV-B as well as UV-A radiation, is based on the hour of maximal UV radiation. Details on the assessment of UV radiation in the SALIA cohort are given in [11].

Genome-wide genotype data

For 586 of the women participating in the second follow-up investigation genome-wide genotyping was performed using the Axiom Precision Medicine Research Array (Affymetrix, Santa Clara, CA, USA). Single nucleotide polymorphisms (SNPs) were imputed on the Haplotype Reference Consortium (HRC) reference panel (r1.1 2016) using Michigan Imputation Server with Minimac4 [12].

We selected SNPs for our analyses which were previously published to be associated with the formation of pigmented spots [13-17] respectively with lung function indices [18]. For the skin aging outcome 42 of the 46 selected SNPs were available in our study. For the lung function outcomes 278 of the 279 lung function associated SNPs published in [18], Supplementary Table 9, were available in our study. Details on the selected SNPs and their availability in our cohort are listed in Supplementary Tables S2 and S3.

Further predictors

We collected information on age, height, weight, urban/rural residence, smoking behavior (current and former smoking, pack-years, environmental tobacco smoke (ETS) at work and at home), socio-economic status (SES; defined as low, medium or high according to the highest number of school years of the participant or her spouse), lack of physical activity (never done sports regularly), hormone replacement therapy (HRT), skin type as well as usage of sunbeds and creams with a sun

protection factor. The ordinal variable SES was dummy-coded with two binary variables SES_{med} and SES_{high}, so that a low SES is the reference category.

The body mass index (BMI) was calculated from height and weight at baseline, first follow-up and second follow-up. Due to many missing values at first follow-up, we calculated the mean of BMI at baseline and BMI at first follow-up (if available) to reflect BMI up to the time of first follow-up.

The participants also completed a food frequency questionnaire. The women's diet was scored according to the Mediterranean diet (MeDi) score of Panagiotakos et al. [19]. Details are given in the following paragraph.

(Modified) Mediterranean diet (MeDi) score

During the second follow-up investigation the participants completed a food frequency questionnaire answering the question "How often do you eat the following food?" for the following items (among others): wholewheat bread, rice, pasta, fruits, raw vegetables and salad, cooked vegetables, potatoes, fish, red meat, poultry and dairy products. Possible answers were "almost daily", "several times per week", "once a week", "several times per month", "once a month or less" and "never".

Missing values in these 11 items (32 values, respectively 0.6%) were imputed using a *k*-nearest neighbor approach for categorical data implemented in the R function knncatimpute from package scrime [20].

We summarized the answers for the items rice, pasta and wholewheat bread in a combined variable "non-refined cereals". Subsequently the items were scored in the style of the Mediterranean diet score of Panagiotakos et al. [19]. Their score is based on 11 categories: non-refined cereals, fruits, vegetables, potatoes, legumes, olive oil, fish, red meat, poultry, full fat dairy products and alcohol. In SALIA, no data on legumes and olive oil were collected, while information for raw vegetables as well as cooked

vegetables was available. Thus, we constructed our Mediterranean diet score based on nine food items: non-refined cereals, fruits, raw vegetables, cooked vegetables, potatoes, fish, red meat, poultry and dairy products. The intake of alcoholic beverages was not included in our modified version of the MeDi score, since it is much more common in Germany to drink beer than wine.

For food items that should be limited according to the Mediterranean diet (red meat, poultry, dairy products), the answers were scored from 0 for “almost daily” up to 5 for “never” (see also Supplementary Table S1). For food items that are recommended according to the Mediterranean diet (non-refined cereals, fruits, raw vegetables, cooked vegetables, potatoes, fish), the answers were scored in reverse order with value 5 for “almost daily” to 0 for “never”.

The final (modified) MeDi score is calculated as the sum of the three items to be limited and the six recommended items and can therefore take values between 0 and 45 with higher values reflecting better adherence to the Mediterranean diet.

Missing values and analysis population

The analyses were restricted to those participants, for which SNP data, as well as the outcome values, were available. Thus, the analysis population for the lung function outcomes includes 510 women, and for the skin aging outcome 547 women.

Since the sample size is quite limited and there were only very few missing values in some covariates, we chose to impute them. Missing values in SES (1 value), ETS at home (1), ETS at work (1), physical activity (2) and HRT (3) were imputed by simple random sampling from the respective marginal empirical distributions. One missing value in pack-years of a former smoking woman was imputed by the median of pack years in the group of former smokers.

Correlation plots to demonstrate the de-correlating effect of building risk scores

A correlation matrix of all predictors except the SNP variables is calculated using Spearman's rank correlation and visualized in a correlation plot with the help of the R package "corrplot" [21]. A respective plot with the correlation matrix of the estimated risk scores in the test sample (averaged across all bootstrap replications) is created after application of the model to illustrate the de-correlating effect of building the risk scores.

The correlation plots for the original predictors (Supplementary Figures 4 and 5) show clear correlation patterns with at least two highly correlated variables for each RS. For the predictors of the final model (single predictors and risk scores) in the test samples, the plots (Supplementary Figures 6 to 8) show only slight to moderate correlations, thus, confirming the de-correlating effect of the statistical approach.

Supplementary Tables

Table S1: Scoring of the food frequencies for the Mediterranean diet score.

	almost daily	several times per week	once a week	several times per month	once a month or less	never
**Items to be limited**						
red meat	0	1	2	3	4	5
poultry	0	1	2	3	4	5
dairy products	0	1	2	3	4	5
**Items recommended**						
non-refined cereals	5	4	3	2	1	0
fruits	5	4	3	2	1	0
raw vegetables	5	4	3	2	1	0
cooked vegetables	5	4	3	2	1	0
potatoes	5	4	3	2	1	0
fish	5	4	3	2	1	0

Maximal adherence score	45
Minimal adherence score	0

Table S2: Single nucleotide polymorphisms used in the genetic risk score for the skin aging trait.

rsID	chromosome	source*	availability in SALIA
rs12693889	2	Shin et al. (2020)	
rs1121878	3	Laville et al. (2016)	
rs13071701	3	Laville et al. (2016)	
rs1491687	3	Laville et al. (2016)	
rs1529585	3	Laville et al. (2016)	
rs1544978	3	Laville et al. (2016)	
rs17006579	3	Laville et al. (2016)	
rs576	3	Laville et al. (2016)	
rs6777363	3	Laville et al. (2016)	
rs704246	3	Laville et al. (2016)	
rs7430957	3	Laville et al. (2016)	
rs7623610	3	Laville et al. (2016)	not available
rs9858495	3	Laville et al. (2016)	
rs183671	5	Liu et al. (2016)	
rs251468	5	Endo et al. (2018)	
rs32579	5	Shin et al. (2020)	
rs12203592	6	Laville et al. (2016), Jacobs et al. (2015)	
rs2524065	6	Laville et al. (2016)	
rs2524067	6	Laville et al. (2016)	
rs2524069	6	Laville et al. (2016)	

rs2844613	6	Laville et al. (2016)	
rs2844614	6	Laville et al. (2016)	
rs2853947	6	Laville et al. (2016)	
rs2853949	6	Laville et al. (2016)	
rs6904500	6	Laville et al. (2016)	
rs9350204	6	Laville et al. (2016)	
rs9358294	6	Laville et al. (2016)	
rs12377342	9	Laville et al. (2016)	
rs16935073	9	Shin et al. (2020)	
rs62543565	9	Jacobs et al. (2015)	
rs643319	9	Shin et al. (2020)	
rs7034903	9	Laville et al. (2016)	
rs11198112	10	Shin et al. (2020)	
rs61866017	10	Endo et al. (2018)	
rs1393350	11	Liu et al. (2016)	
rs912292	13	Laville et al. (2016)	
rs1498519	15	Laville et al. (2016)	
rs17650960	15	Laville et al. (2016)	not available
rs1805005	16	Liu et al. (2016)	
rs1805007	16	Liu et al. (2016)	
rs1805008	16	Liu et al. (2016)	
rs1805009	16	Liu et al. (2016)	not available
rs2228479	16	Shin et al. (2020)	
rs35063026	16	Jacobs et al. (2015)	
rs2240751	19	Shin et al. (2020)	not available
rs6059655	20	Jacobs et al. (2015)	

* Publications found in Pubmed using the search terms 'lentigines GWAS', '"pigmented spots" GWAS' and '"age spots" GWAS'

Table S3: Single nucleotide polymorphisms used in the genetic risk score for the lung function traits.

rsID	chromosome	availability in SALIA	rsID	chromosome	availability in SALIA
rs1008833	1		rs2544536	2	
rs10874851	1		rs2571445	2	
rs10919604	1		rs3791679	2	
rs11165787	1		rs4294980	2	
rs11205354	1		rs4308141	2	
rs1192415	1		rs4674407	2	
rs12096239	1		rs4952564	2	
rs12737805	1		rs55884799	2	
rs12757436	1		rs62168891	2	
rs1338227	1		rs62201738	2	
rs1416685	1		rs6431620	2	
rs141942982	1		rs6435952	2	
rs17009288	1		rs6437219	2	
rs17531405	1		rs6710301	2	
rs2146098	1		rs6733504	2	
rs2355237	1		rs6751968	2	
rs2799098	1		rs72902177	2	
rs2816992	1		rs732990	2	
rs28613267	1		rs7424771	2	
rs35043843	1		rs985256	2	
rs4309038	1		rs12497779	3	
rs4651005	1		rs12634907	3	
rs556648	1		rs12715478	3	
rs6604614	1		rs1529672	3	
rs72673461	1		rs1610265	3	
rs75128958	1		rs17666332	3	
rs755249	1		rs1799807	3	
rs878471	1		rs2974389	3	
rs9435733	1		rs2999090	3	
rs9438626	1		rs35480566	3	
rs9661687	1		rs4132748	3	
rs9661802	1		rs586936	3	
rs9970286	1		rs6445932	3	
rs12470864	2		rs6780171	3	
rs1249096	2		rs73048404	3	
rs12997625	2		rs78101726	3	
rs13009582	2		rs879394	3	
rs13430465	2		rs11098196	4	
rs1406225	2		rs11722225	4	
rs2084448	2		rs12331869	4	
rs2304340	2		rs13109426	4	

rsID	chromosome	availability in SALIA	rsID	chromosome	availability in SALIA
rs13116999	4		rs7752448	6	
rs13141641	4		rs7753012	6	
rs2353940	4		rs9274247	6	not available
rs2609279	4		rs9357446	6	
rs2869966	4		rs9385988	6	
rs34712979	4		rs9472541	6	
rs62289340	4		rs9689096	6	
rs62316310	4		rs12698403	7	
rs6533183	4		rs12707691	7	
rs10059661	5		rs1513272	7	
rs10059996	5		rs17232687	7	
rs11134766	5		rs193686	7	
rs11134789	5		rs2261360	7	
rs11739847	5		rs4318980	7	
rs11952673	5		rs4721442	7	
rs12522114	5		rs4721457	7	
rs17163397	5		rs559233	7	
rs1800888	5		rs62454414	7	
rs2441026	5		rs330939	8	
rs268717	5		rs4128298	8	
rs3843503	5		rs7465401	8	
rs425102	5		rs7838717	8	
rs4866846	5		rs10983184	9	
rs6859730	5		rs1107677	9	
rs7733410	5		rs1491106	9	
rs79898473	5		rs1570203	9	
rs987068	5		rs28446321	9	
rs10498672	6		rs4073153	9	
rs1102077	6		rs57649467	9	
rs12198986	6		rs7024579	9	
rs12202314	6		rs7041139	9	
rs1294417	6		rs72743974	9	
rs13198081	6		rs771662	9	
rs13206405	6		rs967497	9	
rs17280293	6		rs10998018	10	
rs2070600	6		rs11191841	10	
rs2076295	6		rs1259605	10	
rs2627237	6		rs1274475	10	
rs2798641	6		rs2637254	10	
rs2894837	6		rs4279944	10	
rs6918725	6		rs60820984	10	

rsID	chromosome	availability in SALIA	rsID	chromosome	availability in SALIA
rs7082066	10		rs74053129	14	
rs7090277	10		rs12917612	15	
rs7098573	10		rs1441358	15	
rs721917	10		rs1896797	15	
rs7914842	10		rs2012453	15	
rs10836366	11		rs2304645	15	
rs10838435	11		rs34245505	15	
rs11234768	11		rs35251997	15	
rs17596617	11		rs4924525	15	
rs2027761	11		rs56383987	15	
rs541601	11		rs62012772	15	
rs71490394	11		rs62015883	15	
rs10841302	12		rs7176074	15	
rs10850377	12		rs79234094	15	
rs11172113	12		rs11074547	16	
rs11176001	12		rs11648508	16	
rs113745635	12		rs11858992	16	
rs1244869	12		rs12446589	16	
rs12811814	12		rs12918140	16	
rs12825748	12		rs2345443	16	
rs1689510	12		rs35420030	16	
rs2701110	12		rs3751837	16	
rs35505	12		rs56104880	16	
rs56196860	12		rs6539952	16	
rs56390486	12		rs76219171	16	
rs7970544	12		rs78442819	16	
rs7977418	12		rs8047194	16	
rs972936	12		rs11653958	17	
rs9788269	12		rs1215	17	
rs11620380	13		rs12945803	17	
rs2812208	13		rs2244592	17	
rs4885681	13		rs28519449	17	
rs803765	13		rs34351630	17	
rs9533803	13		rs35246838	17	
rs9634470	13		rs4796334	17	
rs10141786	14		rs4968200	17	
rs11160037	14		rs59606152	17	
rs11621587	14		rs62070648	17	
rs1756281	14		rs6501431	17	
rs1951121	14		rs6501455	17	
rs35107139	14		rs77672322	17	

rsID	chromosome	availability in SALIA
rs79412431	17	
rs8068952	17	
rs8069451	17	
rs8082036	17	
rs9892893	17	
rs996865	17	
rs11082051	18	
rs12607758	18	
rs1668091	18	
rs1985511	18	
rs2202572	18	
rs303752	18	
rs513953	18	
rs8089099	18	
rs9807668	18	
rs9947743	18	
rs11085744	19	
rs2967516	19	
rs34093919	19	
rs9636166	19	
rs12481092	20	
rs143384	20	
rs2145272	20	
rs2236180	20	
rs4413223	20	
rs4809221	20	
rs6032942	20	
rs12627254	21	
rs62213732	21	
rs113111175	22	
rs1978968	22	
rs2283847	22	
rs9610955	22	

Supplementary Figures

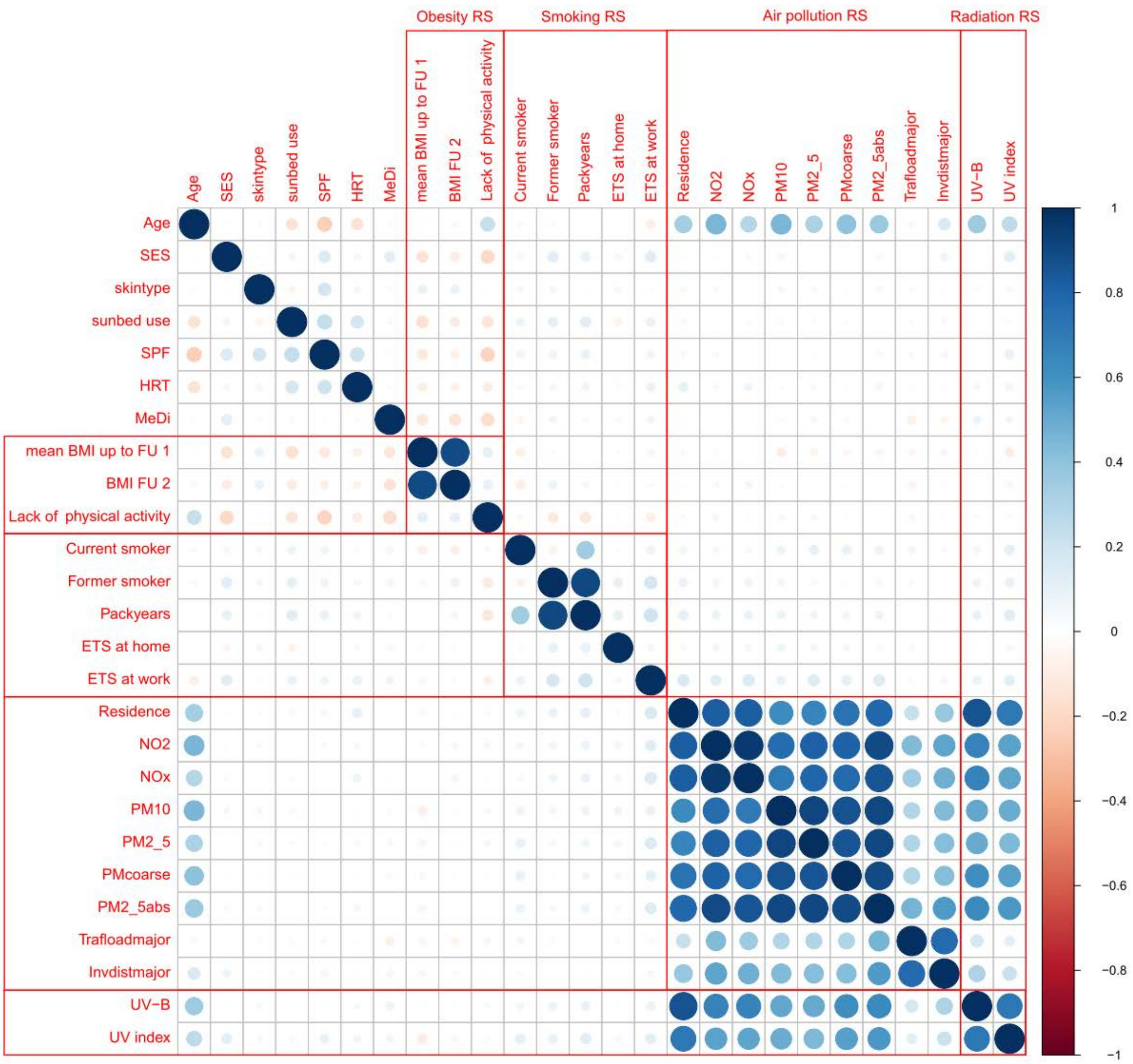

Figure S1: Correlation plot of the original variables used in the skin aging analysis (excluding the SNP variables).

BMI: body mass index; ETS: environmental tobacco smoke; FU: follow-up; HRT: hormone replacement therapy; Invdistmajor: inverse distance to next major road (>5000 vehicles / day); MeDi: Mediterranean diet; NO_2: nitrogen dioxide; NO_x: nitrogen oxides; PM_{10}: particulate matter with aerodynamic diameter <= 10 µm; $PM_{2.5}$: particulate matter with aerodynamic diameter <= 2.5 µm; PM_{coarse}: coarse fraction of PM_{10} calculated as PM_{10} minus $PM_{2.5}$; $PM_{2.5abs}$: absorbance of particulate matter with aerodynamic diameter of <= 2.5 µm; RS: risk score; SES: socio-

economic status; SNP: single nucleotide polymorphism; SPF: sun protection factor; Trafloadmajor: total traffic load (number of vehicles / day * length of road segments) from major roads (>5000 vehicles / day) within 100m buffer; UV: ultraviolet.

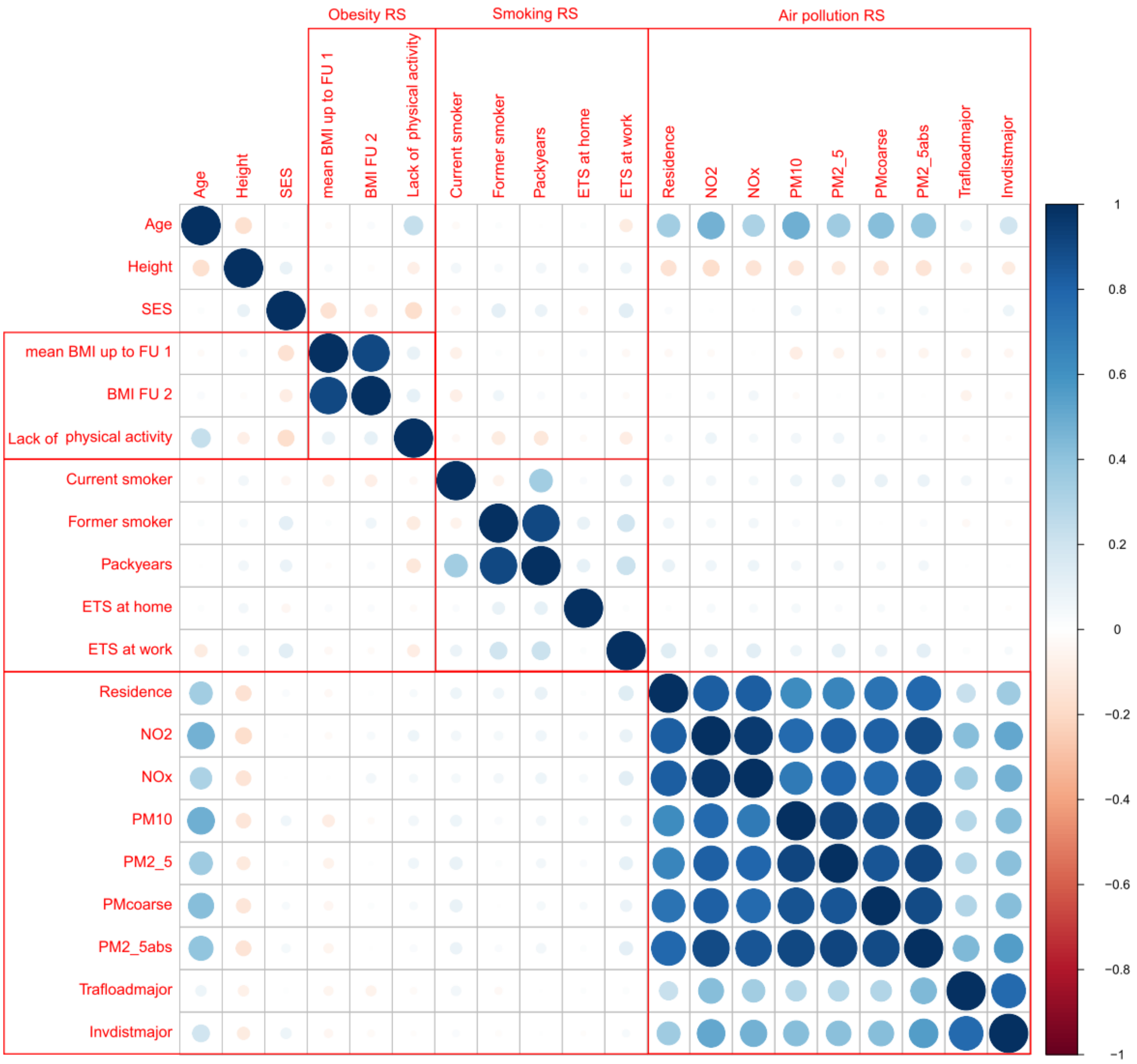

Figure S2: Correlation plot of the original variables used in the lung function analysis (excluding the SNP variables).

BMI: body mass index; ETS: environmental tobacco smoke; FU: follow-up; Invdistmajor: inverse distance to next major road (>5000 vehicles / day); NO_2: nitrogen dioxide; NO_x: nitrogen oxides; PM_{10}: particulate matter with aerodynamic diameter <= 10 µm; $PM_{2.5}$: particulate matter with aerodynamic diameter <= 2.5 µm;

PM_{coarse}: coarse fraction of PM_{10} calculated as PM_{10} minus $PM_{2.5}$; $PM_{2.5abs}$: absorbance of particulate matter with aerodynamic diameter of <= 2.5 µm; RS: risk score; SES: socio-economic status; SNP: single nucleotide polymorphism; Trafloadmajor: total traffic load (number of vehicles / day * length of road segments) from major roads (>5000 vehicles / day) within 100m buffer.

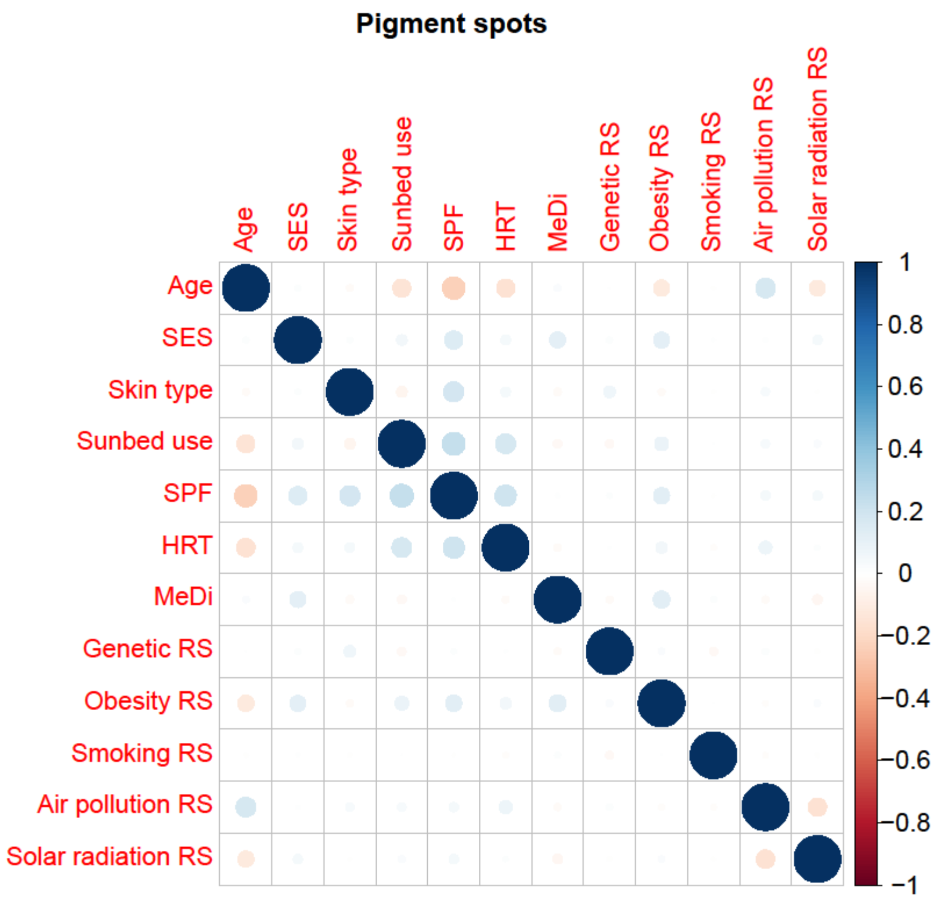

Figure S3: Correlation plot of the risk scores and single predictors used in the skin aging analysis.

HRT: hormone replacement therapy; MeDi: Mediterranean diet; RS: risk score; SES: socio-economic status; SPF: sun protection factor.

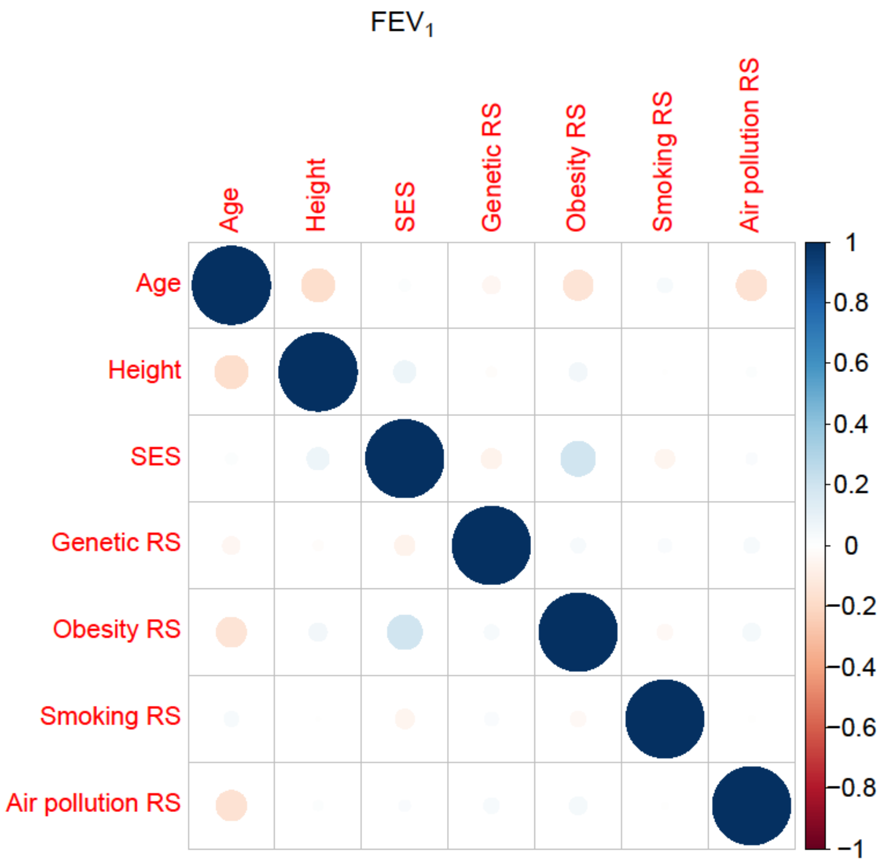

Figure S4: Correlation plot of the risk scores and single predictors used in the analysis of FEV1.

FEV_1: forced expiratory volume in 1 second; RS: risk score; SES: socio-economic status.

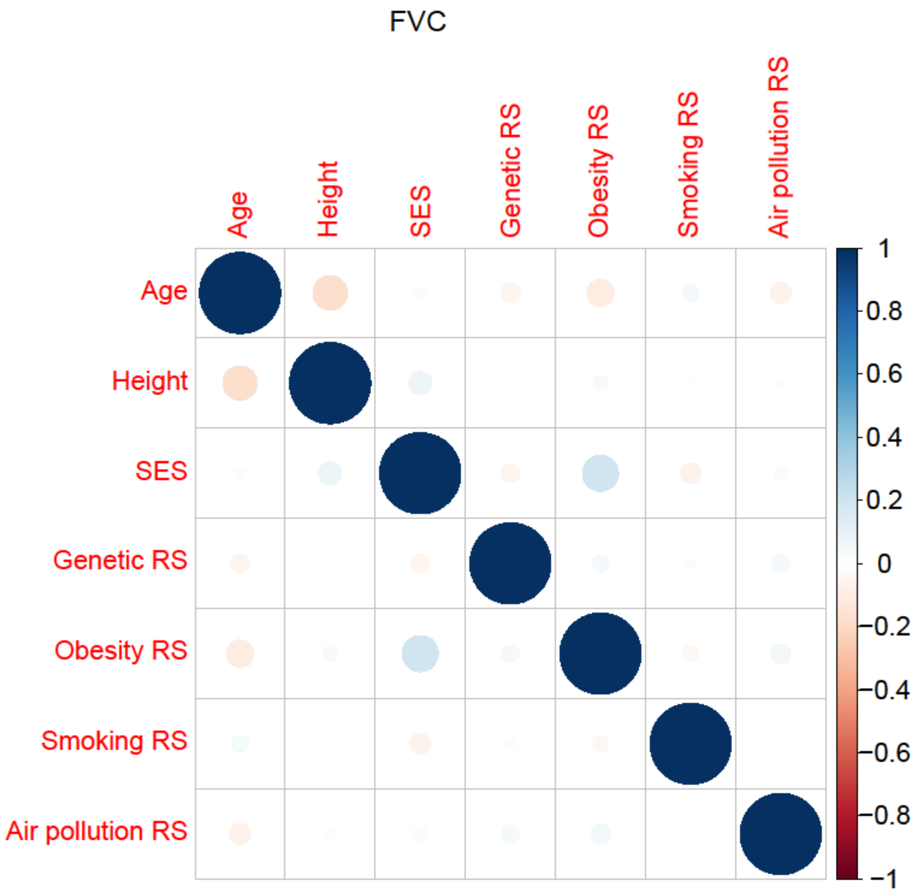

Figure S5: Correlation plot of the risk scores and single predictors used in the analysis of FVC.

FVC: forced vital capacity; RS: risk score; SES: socio-economic status.

References

1. Schikowski, T.; Sugiri, D.; Ranft, U.; Gehring, U.; Heinrich, J.; Wichmann, H.E.; Krämer, U. Long-term air pollution exposure and living close to busy roads are associated with COPD in women. *Respir Res* **2005**, *6*, 152-162, doi:10.1186/1465-9921-6-152.
2. Schikowski, T.; Sugiri, D.; Ranft, U.; Gehring, U.; Heinrich, J.; Wichmann, H.E.; Krämer, U. Does respiratory health contribute to the effects of long-term air pollution exposure on cardiovascular mortality? *Respir Res* **2007**, *8*, 20, doi:10.1186/1465-9921-8-20.
3. Schikowski, T.; Vossoughi, M.; Vierkötter, A.; Schulte, T.; Teichert, T.; Sugiri, D.; Fehsel, K.; Tzivian, L.; Bae, I.S.; Ranft, U.; et al. Association of air pollution with cognitive functions and its modification by APOE gene variants in elderly women. *Environ Res* **2015**, *142*, 10-16, doi:10.1016/j.envres.2015.06.009
4. Vierkötter, A.; Ranft, U.; Krämer, U.; Sugiri, D.; Reimann, V.; Krutmann, J. The SCINEXA: A novel, validated score to simultaneously assess and differentiate between intrinsic and extrinsic skin ageing. *J. Dermatol. Sci* **2009**, *53*, 207-211.
5. Tschachler, E.; Morizot, F. Ethnic differences in skin aging. In *Skin Aging*, Krutmann, J., Gilchrest, B.A., Eds.; Springer Verlag GmbH: Berlin, 2006.
6. Miller, M.R.; Hankinson, J.; Brusasco, V.; Burgos, F.; Casaburi, R.; Coates, A.; Crapo, R.; Enright, P.; van der Grinten, C.P.; Gustafsson, P.; et al. Standardisation of spirometry. *Eur. Respir. J* **2005**, *26*, 319-338.
7. Quanjer, P.H.; Stanojevic, S.; Cole, T.J.; Baur, X.; Hall, G.L.; Culver, B.H.; Enright, P.L.; Hankinson, J.L.; Ip, M.S.M.; Zheng, J.; et al. Multi-ethnic reference values for spirometry for the 3–95-yr age range: the global lung function 2012 equations. *Eur Respir J* **2012**, *40*, 1324-1343, doi:10.1183/09031936.00080312.

8. Beelen, R.; Hoek, G.; Vienneau, D.; Eeftens, M.; Dimakopoulou, K.; Pedeli, X.; Tsai, M.-Y.; Künzli, N.; Schikowski, T.; Marcon, A.; et al. Development of NO_2 and NO_x land use regression models for estimating air pollution exposure in 36 study areas in Europe - The ESCAPE project. *Atmos Environ* **2013**, *72*, 10-23, doi:10.1016/j.atmosenv.2013.02.037.
9. Eeftens, M.; Beelen, R.; de Hoogh, K.; Bellander, T.; Cesaroni, G.; Cirach, M.; Declercq, C.; Dedele, A.; Dons, E.; de Nazelle, A.; et al. Development of land use regression models for $PM_{2.5}$, $PM_{2.5}$ absorbance, PM_{10} and PM_{coarse} in 20 European study areas;results of the ESCAPE project. *Environ Sci Technol* **2012**, *46*, 11195-11205, doi:10.1021/es301948k.
10. Adam, M.; Schikowski, T.; Carsin, A.E.; Cai, Y.; Jacquemin, B.; Sanchez, M.; Vierkötter, A.; Marcon, A.; Keidel, D.; Sugiri, D.; et al. Adult lung function and long-term air pollution exposure. ESCAPE: a multicentre cohort study and meta-analysis. *Eur Resp J* **2014**, erj01300-02014, doi:10.1183/09031936.00130014.
11. Hüls, A.; Sugiri, D.; Fuks, K.; Krutmann, J.; Schikowski, T. Lentigine Formation in Caucasian Women—Interaction between Particulate Matter and Solar UVR. *J Invest Dermatol* **2019**, *139*, 974-976, doi:10.1016/j.jid.2018.09.034.
12. Das, S.; Forer, L.; Schönherr, S. Next-generation genotype imputation service and methods. *Nature Genet* **2016**, *48*, 1284–1287, doi:10.1038/ng.3656.
13. Endo, C.; Johnson, T.A.; Morino, R.; Nakazono, K.; Kamitsuji, S.; Akita, M.; Kawajiri, M.; Yamasaki, T.; Kami, A.; Hoshi, Y.; et al. Genome-wide association study in Japanese females identifies fifteen novel skin-related trait associations. *Sci Rep* **2018**, *8*, 8974, doi:10.1038/s41598-018-27145-2.
14. Jacobs, L.C.; Hamer, M.A.; Gunn, D.A.; Deelen, J.; Lall, J.S.; van Heemst, D.; Uh, H.W.; Hofman, A.; Uitterlinden, A.G.; Griffiths, C.E.M.; et al. A Genome-Wide Association Study Identifies the Skin Color Genes IRF4, MC1R, ASIP, and BNC2 Influencing Facial Pigmented Spots. *J Invest Dermatol* **2015**, *135*, 1735-1742, doi:10.1038/jid.2015.62.
15. Laville, V.; Clerc, S.L.; Ezzedine, K.; Jdid, R.; Taing, L.; Labib, T.; Coulonges, C.; Ulveling, D.; Carpentier, W.; Galan, P.; et al. A genome-wide association study in Caucasian women suggests the involvement of HLA genes in the severity of facial solar lentigines. *Pigment Cell & Melanoma Res* **2016**, *29*, 550-558, doi:10.1111/pcmr.12502.
16. Liu, F.; Hamer, M.A.; Deelen, J.; Lall, J.S.; Jacobs, L.; van Heemst, D.; Murray, P.G.; Wollstein, A.; de Craen, A.J.; Uh, H.W.; et al. The MC1R Gene and Youthful Looks. *Curr Biol* **2016**, *26*, 1213-1220, doi:10.1016/j.cub.2016.03.008.
17. Shin, J.-G.; Leem, S.; Kim, B.; Kim, Y.; Lee, S.-G.; Song, H.J.; Seo, J.Y.; Park, S.G.; Won, H.-H.; Kang, N.G. GWAS Analysis of 17,019 Korean Women Identifies the Variants Associated with Facial Pigmented Spots. *J Invest Dermatol* **2021**, *141*, 555-562, doi:10.1016/j.jid.2020.08.007.
18. Shrine, N.; Guyatt, A.L.; Erzurumluoglu, A.M.; Jackson, V.E.; Hobbs, B.D.; Melbourne, C.A.; Batini, C.; Fawcett, K.A.; Song, K.; Sakornsakolpat, P.; et al. New genetic signals for lung function highlight pathways and chronic obstructive pulmonary disease associations across multiple ancestries. *Nature Genet* **2019**, *51*, 481-493, doi:10.1038/s41588-018-0321-7.
19. Panagiotakos, D.B.; Pitsavos, C.; Arvaniti, F.; Stefanadis, C. Adherence to the Mediterranean food pattern predicts the prevalence of hypertension, hypercholesterolemia, diabetes and obesity, among healthy adults; the accuracy of the MedDietScore. *Prev Med* **2007**, *44*, 335-340, doi:10.1016/j.ypmed.2006.12.009.
20. Schwender, H. *scrime: Analysis of High-Dimensional Categorical Data Such as SNP Data*, R package version 1.3.5; 2018.
21. Wei, T.; Simko, V. *R package "corrplot": Visualization of a Correlation Matrix*, R package version 0.84; 2017.
